# Induction of CX3CL1 expression by LPS and its impact on invasion and migration in oral squamous cell carcinoma

**DOI:** 10.3389/fcell.2024.1371323

**Published:** 2024-06-10

**Authors:** Chanjuan He, Yuehan Wu, Xiaoxu Nan, Weifang Zhang, Yu Luo, Honglan Wang, Mengqi Li, Changyue Liu, Jiaming Liu, Xuelin Mou, Ying Liu

**Affiliations:** ^1^ Department of Stomatology, Affiliated Hospital of North Sichuan Medical College, Nanchong, China; ^2^ Changsha Stomatological Hospital, Changsha, China; ^3^ Department of Stomatology, North Sichuan Medical College, Nanchong, China; ^4^ Hunan Prevention and Treatment Institute for Occupational Diseases, Changsha, China

**Keywords:** oral squamous cell carcinoma(OSCC), CX3CL1, CX3CR1, lipopolysaccharide(LPS), epithelial-mesenchymal transition (EMT)

## Abstract

**Purpose:** This study aimed to explore the expression of *CX3CL1* induced by lipopolysaccharide (LPS) in oral squamous cell carcinoma (OSCC) and its impact on biological characteristics such as invasion and migration, taking the foundation for new targets for the treatment and prognosis of OSCC.

**Methods:** This study utilized a variety of techniques, including bioinformatics, molecular biology, and cell experiments, to investigate the expression of *CX3CL1* and its receptor *CX3CR1* in OSCC patients’ cancer tissues or OSCC cell lines. Extracting, organizing, and analyzing the TCGA database on the expression of *CX3CL1* and its receptor *CX3CR1* in cancer tissues and corresponding paracancerous normal tissues of OSCC patients by bioinformatics methods. The expression of *CX3CL1* in cancerous and normal tissues of OSCC patients was verified by IHC, and the changes in mRNA and protein expression of *CX3CL1* and its receptor *CX3CR1* in OSCC cell lines were detected before and after lipopolysaccharide LPS stimulation by RT-PCR, ELISA, and WB. Changes in cell biological behavior by overexpression of *CX3CL1* in OSCC cell lines were detected by CCK-8, Transwell, scratch healing assay, and cloning assay. The effects of overexpressing cell lines on the AKT pathway and Epithelial-mesenchymal Transition (EMT)-related protein expression before and after LPS stimulation were detected by Western Blot.

**Results:** (1) *CX3CL1* and its receptor *CX3CR1* were found to be downregulated in OSCC tissues of patients or OSCC cell lines. (2) After LPS stimulation, *CX3CL1* gene expression increased in both OSCC cell lines, while *CX3CR1* expression remained unchanged. (3) OSCC cell lines overexpressing *CX3CL1* showed changes in cell biological characteristics, including decreased proliferation, invasion, migration, and stemness, which were more pronounced after LPS stimulation. (4) Overexpression of *CX3CL1* in OSCC cell lines decreased EMT-related protein expression and AKT phosphorylation. On the contrary were promoted by LPS stimulation.

**Conclusion:** *CX3CL1* and *CX3CR1* are downregulated in OSCC cancer tissues and cell lines compared to adjacent normal tissues and cells. LPS stimulation increases *CX3CL1* expression in OSCC cell lines, suggesting that inflammation may induce *CX3CL1* expression and that the *CX3CL1* gene may play an important role in OSCC progression. Overexpression of *CX3CL1* inhibits OSCC cell proliferation, migration, invasion, and stemness, suggesting that *CX3CL1* plays a critical role in suppressing OSCC development. *CX3CL1* suppresses OSCC invasion and migration by affecting EMT progression and AKT phosphorylation, and partially reverse the process that LPS causes and affects the development of OSCC.

## 1 Introduction

Oral squamous cell carcinoma (OSCC) is one of the most common cancers of the oral and maxillofacial, and is considered one of the most prevalent cancers worldwide. It is characterized by high incidence, strong recurrence ability, and low 5-year survival rate, making it a significant public health issue worldwide (Bray et al., 2018; Madhura et al., 2020; Ling et al., 2021). Although surgical treatment with adjuvant radiotherapy or chemotherapy remains the mainstay of therapy, it can cause irreversible damage to patients’ quality of life and psychological health. Therefore, studying and understanding the mechanisms and processes of OSCC can help identify new genetic targets, and is of great significance for improving the treatment and survival rates of OSCC.

The occurrence and development of OSCC are closely related to complex genetic and environmental factors. Research has shown that inflammation is caused by the body’s immune response to foreign substances, and that patients with OSCC have a large number of pathogenic microorganisms in their oral cavity. When the microenvironment is disrupted, it may have an impact on the occurrence and development of OSCC (He et al., 2015; Zhang et al., 2019; Kakabadze et al., 2020; Kong et al., 2020). Lipopolysaccharide (LPS) is the main component of the cell wall and endotoxin of Gram-negative bacteria, and is an important molecule in bacterial pathogenesis. Numerous studies have shown that LPS plays an important role in promoting invasion and migration of OSCC (Huang et al., 2017; Oliveira et al., 2017; Ye et al., 2021; Omori et al., 2024). Our previous studies have shown that LPS stimulates the NLRP3/Caspase-1 inflammatory signaling pathway to promote the proliferation and migration of oral squamous cell cancer cells (He et al., 2023).

Inflammation is a process that is caused by a local or systemic immune response involving many cytokines, immune cells, and other factors. Chemokines are a class of proteins secreted by various types of cells that have functions such as chemotaxis (Brennan et al., 2024), regulating leukocyte migration and homing, and participating in inflammatory and immune regulatory processes. They play an especially important role in inflammation control and maintaining homeostasis in the body (Bhatia and Burtness, 2023). Chemokines and their receptors also play important roles in the occurrence and development of various types of cancers, such as cervical cancer (Dreyer et al., 2021) and colorectal cancer (Garrido et al., 2021), and some chemokines are closely related to the poor clinical prognosis of various cancers.

*CX3CL1*, also known as Fractalkine, is the only member of the CX3C chemokine family, and its only receptor is CX3CR1. It has two forms: membrane-bound and soluble, which mediate different biological activities. The membrane-bound form enhances cell adhesion, while soluble *CX3CL1* has the function of chemotaxis towards cells expressing *CX3CR1*. *CX3CL1* is the only member of the CX3C chemokine subfamily and has been shown to have anti-cancer effects in other tumors such as colon cancer (Hughes and Nibbs, 2018), breast cancer (Kadomoto et al., 2020), and cervical cancer (Khare et al., 2021). It also has important implications in regulating inflammation and cancer metastasis. However, the role of *CX3CL1* in OSCC is not well understood and has been subject to controversy.*CX3CL1* exhibits markedly different effects in different types of tumors, especially in OSCC, where research is limited and whether it promotes or inhibits cancer remains unclear (Li et al., 2020; Kavarthapu and Gurumoorthy, 2021).

Currently, there are some studies on LPS and other types of tumors, but there has been no research that combines LPS, CX3CL1-CX3CR1 axis, and oral cancer. Therefore, it is crucial to explore the relationship between LPS, *CX3CL1*, and OSCC, which is important for early diagnosis, treatment, prognosis prediction, improving the quality of life for patients, and increasing the 5-year survival rate. This research also provides a new perspective for exploring the development of OSCC.

## 2 Materials and methods

### 2.1 Bioinformatics analysis

The genetic data related to cancer tissues and adjacent tissues of oral squamous cell carcinoma (OSCC) patients were downloaded from the TCGA database (GDC(cancer.gov)), and the expression profiles of *CX3CL1* and *CX3CR1* in cancer tissues and corresponding adjacent tissues of OSCC patients were constructed through data analysis.

### 2.2 Patients, specimens and tissue collection

We collected clinical and pathological specimens from 25 patients who underwent radical surgery for OSCC in the Department of Oral and Maxillofacial Surgery at the Affiliated Hospital of North Sichuan Medical College between September 2020 and December 2022, after excluding and including according to strict criteria. Immediately after tumor resection, OSCC tissues of 0.5 cm–1 cm size without necrosis were excised from the tumor site, and adjacent normal tissues (ANTs) of 0.5 cm–1 cm size were collected from areas more than 3 cm away from the tumor margin. These tissues were aliquoted into enzyme-free EP tubes, labeled, and rapidly cooled in liquid nitrogen before being stored at −80°C.The study was approved by the Ethics Committee of the Affiliated Hospital of North Sichuan Medical College (ethics approval number: 2022ER111-1).

### 2.3 Cell culture

The OSCC cell lines HSC3, SCC25, CAL27 and UM1, as well as the normal oral mucosal epithelial cell line HOK, were used in this study and authenticated by Short Tandem Repeat (STR) analysis. These cells were cultured in a humidified incubator at 37°C and 5% CO_2_.

### 2.4 Stimulation of OSCC cell lines with lipopolysaccharide (LPS)

When the cell growth reached 70%–80% confluence, the cells were extracted and counted as described above, and then seeded. After the cells adhered to the culture dish, LPS was added. LPS was diluted to 1 μg/mL and 10 μg/mL using complete culture medium, and the cell lines were stimulated for 6 h, 12 h, 24 h, 36 h, and 48 h. The cells were harvested for subsequent experiments.

### 2.5 qRT-PCR

The cell pellets were lysed and total RNA was extracted using TRIzol reagent. The extracted RNA was then reverse transcribed into cDNA using HiScript II Q RT SuperMix from Vazyme (Beijing, China). Subsequently, quantitative real-time polymerase chain reaction (qPCR) analysis was conducted using ChamQ™ SYBR Color qPCR Master Mix (Vazyme). The specific sequences of primers used for the qPCR analysis can be found in Table 1. The qPCR results were analysed using the 2^−ΔΔCT^ method. Each sample was run in triplicate and the experiment was repeated three times independently. The primer sequences are listed in Table 1 below.

**TABLE 1:** Primer sequences.

Genes	Forward primer	Reverse primer
CX3CL1	F:ACC ACG GTG TGA CGA AAT G	R:TGT TGA TAG TGG ATG AGC AAA GC
CX3CR1	F:ACT TTG AGT ACG ATG ATT TGG CT	R:GGT AAA TGT CGG TGA CAC TCT T
β-actin	F:GAG CTA CGA GCT GCC TGA CG	R:GTA GTT TCG TGG ATG CCA CAG

### 2.6 Enzyme-linked immunosorbent assay (ELISA)

50 μL of standard solution and the test sample were added to each well of a 96-well plate, followed by the addition of 50 μL of antibody cocktail solution. The plate was covered with a sealing film and incubated for 1 h at room temperature in a dark and shaking incubator at 400 rpm. Each well was washed three times with 1 × washing buffer PT. The liquid in each well was aspirated, and 100 μL of TMB substrate chromogenic reagent was added to each well. The plate was then incubated at room temperature on a shaker in the dark for 10 min. Finally, 100 μL of stop solution was added to each well, mixed gently, and the OD value was recorded at a wavelength of 450 nm using a microplate reader. ELISA kit manufacturer (ZCIBIO, China).

### 2.7 Western Blot (WB)

The tissue samples were taken out from −80°C freezer and placed on ice. The tissues were cleaned and homogenized in cell lysis buffer. After thorough shaking and centrifugation, the supernatant was collected and the protein concentration was determined using the BCA (Solarbio, Beijing, China) protein assay kit. Protein samples were separated by electrophoresis and transferred to a PVDF membrane, which was then blocked. The membrane was incubated with primary antibodies including *CX3CL1* rabbit polyclonal antibody (1:2000, DF12376, Affinity), *CX3CR1* rabbit polyclonal antibody (1:2000, DF7096, Affinity), E-cadherin mouse monoclonal antibody (1:2000, U3254, Santa Cruz), N-cadherin rabbit polyclonal antibody (1:500 ER0503,HuaBio), Vimentin rabbit polyclonal antibody (1:1000, R1308-6, HuaBio), Snail rabbit polyclonal antibody (1:500 ER1706-22,HuaBio), AKT rabbit polyclonal antibody (1:1000 9272S, CST) and p-AKT rabbit polyclonal antibody (1:1000 9271S, CST)overnight at 4°C. After washing with TBST for three times (10 min each time), the membrane was incubated with appropriate secondary antibodies for 2 h, followed by washing for three times. The protein bands on the PVDF membrane were visualized by exposure and the gray values were analyzed using ImageJ.

### 2.8 Cell proliferation (cell counting Kit-8, CCK-8)

Cells with good growth status and fusion degree of 70%–80% were counted, and 2000 cells were evenly seeded in each well of a 96-well plate. Four replicate wells were set for each group. After the cells adhered to the wall, the medium was replaced and LPS and the same volume of sterile PBS were added, and the cells were incubated for different time periods. After different stimulation times, 10 μL of CCK-8 reagent was added to each well and incubated in a 37°C, 5% CO_2_, saturated humidity incubator for 2 h. The OD value of each well was detected at a wavelength of 450 nm using an ELISA reader.

### 2.9 Scratch wound healing assay

To measure cell migration, 5 × 10^5^ cells were seeded in each well of a 6-well plate and allowed to form a monolayer. A plastic micropipette tip was used to create a scratch wound, and the wound area was immediately recorded. During this period, serum-free medium was used for culture, and the gap area was recorded 24 h and 36 h after scratching using an inverted microscope. ImageJ software was used to quantify changes in gap area.

### 2.10 Transwell

Preparing ECM gel by diluting it in serum-free medium at a ratio of 1:5, the diluted ECM gel was added to the upper chamber and incubated in a humidified incubator for 2 h. Meanwhile, cells with 70%–80% confluence and starved for 12 h were counted, resuspended in serum-free medium, and added to the upper chamber. Complete medium was added to the lower chamber. After incubation for 36 h or 48 h, cells were fixed with formalin, washed, dried, and stained. After washing with PBS for 3 times and drying, images were captured.

### 2.11 Clonal assay

In a six-well plate with a diameter of 35 mm, 500 cells per well were added for cloning assay. The cells were incubated for a period of time and the number of cloned cells over 50 cells was counted using ImageJ software.

### 2.12 Flow cytometry cell cycle experiment

Collect cells with a fusion rate of 70%–80% by centrifugation, resuspend in PBS, and take 1 × 10^6^ cells per well for the flow cytometry cell cycle experiment. Remove the supernatant after centrifugation and resuspend in freshly prepared 70% ice-cold ethanol for fixation at 4°C overnight for 24 h. Remove the supernatant after centrifugation at 1000 rpm for 5 min and mix with the working solution prepared by adding Rnase A and PI staining solution from the flow cytometry cell cycle kit. Analyze the cell staining results at 488 nm red fluorescence using a flow cytometer.

### 2.13 Statistical analysis

The experiments were independently repeated three times, and statistical analysis was performed using GraphPad Prism 8.0. One-way ANOVA, *t*-test, or non-parametric test was used to determine significance, with *p* < 0.05 considered statistically significant.

## 3 Result

### 3.1 Expression of *CX3CL1* and *CX3CR1* genes in oral squamous cell carcinoma (OSCC) tissue and cell lines

The expression of *CX3CL1* and its receptor *CX3CR1* in OSCC was validated through bioinformatics analysis, immunohistochemistry, PCR, ELISA, and WB.

#### 3.1.1 Expression of *CX3CL1* and *CX3CR1* proteins in OSCC tissue was validated by bioinformatics analysis and immunohistochemistry

**FIGURE 1:**
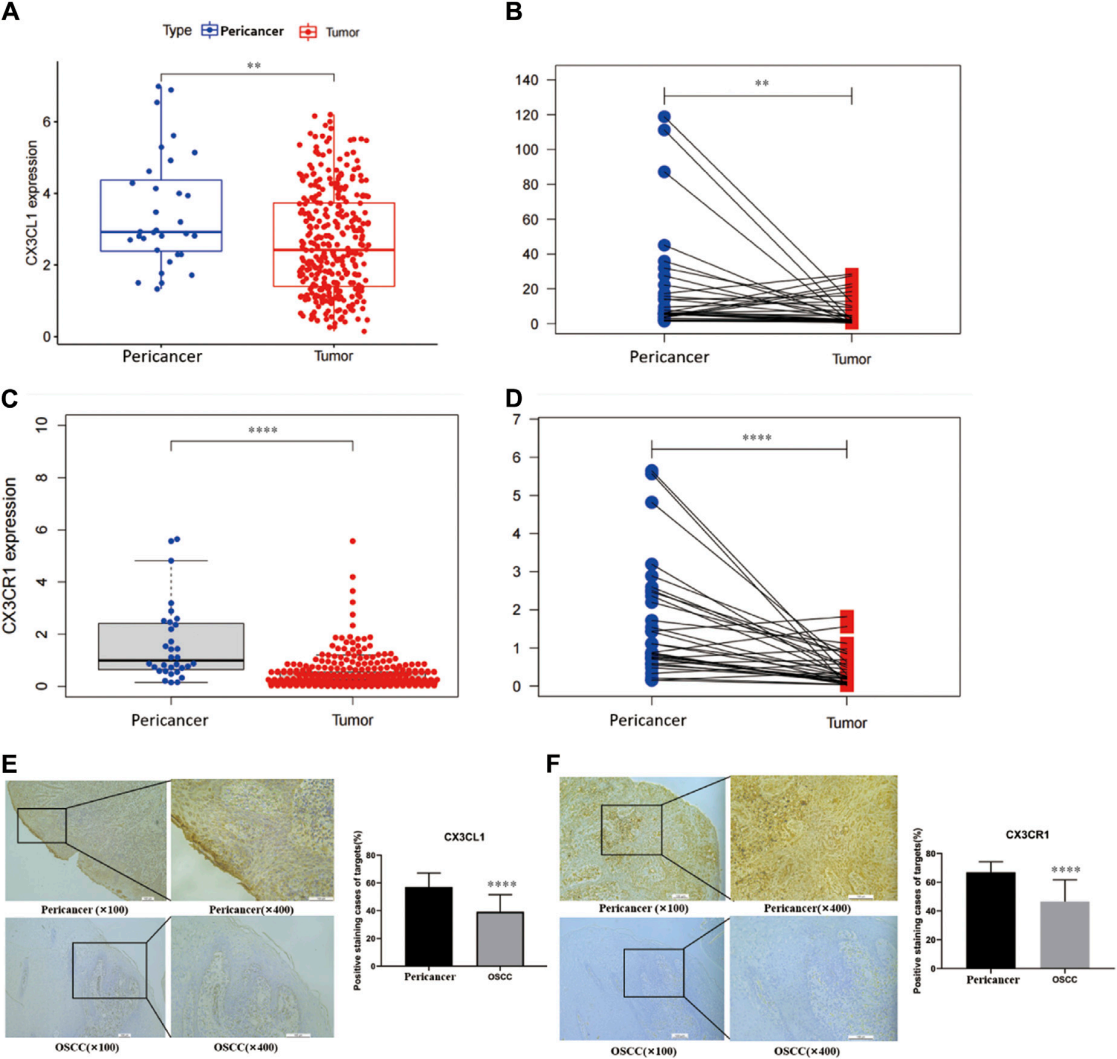
Expression analysis of CX3CL1 and CX3CR1 in OSCC and pericancerous tissues using bioinformatics and immunohistochemistry. Notes: **(A)** and **(C)** show the expression levels of *CX3CL1* and *CX3CR1* in OSCC and pericancerous tissues analyzed by bioinformatics. **(B)** and **(D)** show the relative expression levels of *CX3CL1* and *CX3CR1 i*n OSCC and pericancerous tissues of patients analyzed by bioinformatics. The results indicate that the expression of *CX3CL1* and *CX3CR1* is decreased in OSCC compared to pericancerous tissues. Pericancerous tissues are those surrounding tissues in which no cancerous cells are found on pathologic examination, and tumor refers to OSCC tissues. Immunohistochemical staining and corresponding scoring of *CX3CL1* **(E)** and *CX3CR1* **(F)** were performed in 25 OSCC patients and pericancerous tissues. The yellow or brown staining was mainly located at the basal cell membrane and connective tissue of the epithelial cells. Results showed that the expression levels of *CX3CL1* and *CX3CR1* were significantly lower in OSCC tissues compared to pericancerous tissues. ns p > 0.05; *p < 0.05; **p < 0.01; ***p < 0.001; ****p < 0.0001. Pericancer, pericancerous tissues; Tumor, OSCC tissue.

Bioinformatics analysis showed that *CX3CL1* gene expression was significantly lower in OSCC (tissue from 520 patients) compared to pericancerous tissues (tissue from 44 patients) (Figure 1A), and this difference was also observed in OSCC patients in the TCGA database (Figure 1B). Similarly, bioinformatics analysis showed that *CX3CR1* expression was significantly lower in OSCC compared to pericancerous tissues (Figure 1C), and this difference was also observed in OSCC patients and their corresponding pericancerous tissues (Figure 1D). These findings were further validated through immunohistochemistry.

Through immunohistochemistry, we verified the downregulation of *CX3CL1* and *CX3CR1* genes in OSCC tissues compared to pericancerous tissues. The yellow or light brown staining was mainly located on the basal cell membrane and connective tissue of epithelial cells (Figure 1E). Similarly, the immunohistochemical analysis showed that *CX3CR1* was primarily located on the cell membrane and connective tissue of epithelial cells, and its expression was decreased in OSCC tissues compared to pericancerous tissues (Figure 1F).

#### 3.1.2 *CX3CL1* and *CX3CR1* gene expression of mRNA and protein in OSCC cell lines

**FIGURE 2:**
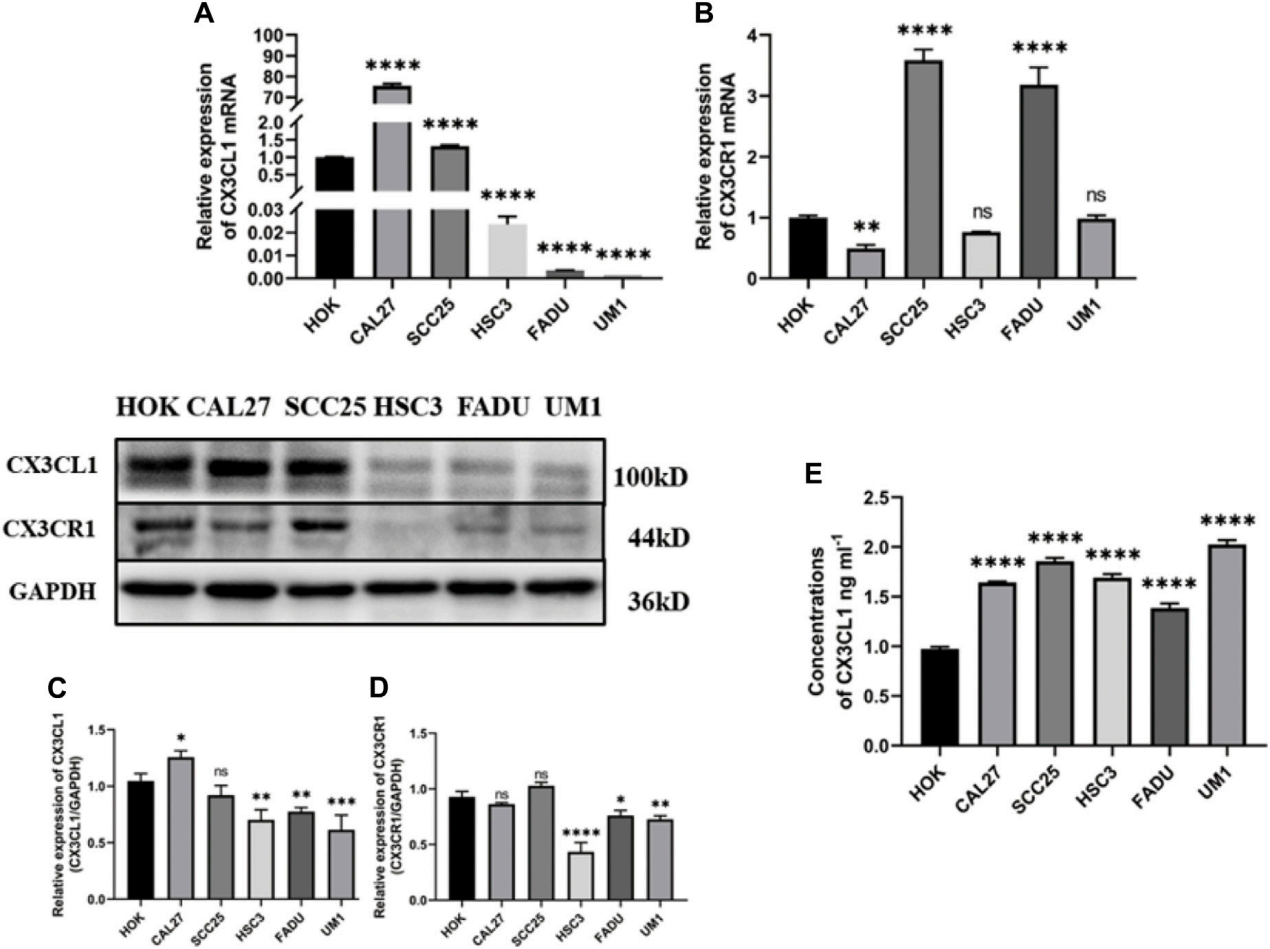
mRNA and protein expression of *CX3CL1* and *CX3CR1* in OSCC cell lines. Notes: **(A)** and **(B)** show the mRNA expression levels of *CX3CL1* and *CX3CR1* in HOK and various OSCC cell lines using PCR; **(C)** and **(D)** show the protein expression levels of membrane-bound *CX3CL1* and *CX3CR1* in HOK and various OSCC cell lines using WB, along with their corresponding gray value analysis; **(E)** shows the OD value analysis of soluble *CX3CL1* protein expression in HOK and various OSCC cell lines using ELISA. The results indicate that *CX3CL1* mRNA and protein expression levels are significantly decreased in most OSCC cell lines compared to HOK, while soluble *CX3CL1* that has the function of chemoattracting cells expressing *CX3CR1*. Expression is increased and *CX3CR1* protein content is significantly decrease. Data were shown as mean with SD (n ≥ 3). *p < 0.05, **p < 0.01, ***p < 0.001, ****p < 0.0001, ns p > 0.05. The inconsistent results between PCR and WB may be due to differences in transcription and translation control. The low expression levels of *CX3CL1* and *CX3CR1* genes in OSCC, as validated by mRNA and protein content analysis in OSCC tissues and cell lines, suggest that they may play a suppressive role in the development and progression of OSCC.

The mRNA and protein expression of *CX3CL1* and *CX3CR1* genes in OSCC cell lines were validated by PCR, ELISA, and WB. PCR analysis showed different mRNA expression patterns of *CX3CL1* and *CX3CR1* genes in various cell lines compared to the normal oral keratinocyte cell line HOK. Except for CAL27 and SCC25, *CX3CL1* mRNA was downregulated in OSCC cell lines (Figure 2A), while *CX3CR1* mRNA was downregulated in all OSCC cell lines except for SCC25 and FADU (Figure 2B).

Furthermore, WB analysis revealed decreased protein expression of membrane-bound *CX3CL1* and *CX3CR1* genes in OSCC cell lines (Figures 2C,D), while ELISA showed an upregulation of soluble *CX3CL1* protein expression in OSCC cell lines with significant differences (Figure 2E). The inconsistent results between PCR and WB may be attributed to the control of transcription and translation processes. As proteins are the ultimate executors of functions, the validated mRNA and protein expression levels of *CX3CL1* and *CX3CR1* genes in OSCC tissue and cell lines suggest their suppressive role in OSCC development and progression.

### 3.2 Impact of lipopolysaccharide (LPS) on the expression of *CX3CL1* and *CX3CR1* genes in OSCC cell lines

The present study investigated the effect of LPS on the mRNA and protein expression of *CX3CL1* and *CX3CR1* genes in OSCC cell lines CAL27 and UM1. The cells were treated with 1 μg/mL and 10 μg/mL LPS for 6 h, 12 h, 24 h, 36 h, and 48 h, and the mRNA and protein expression changes of *CX3CL1* and *CX3CR1* genes were analyzed using PCR, ELISA, and western blotting.

**FIGURE 3:**
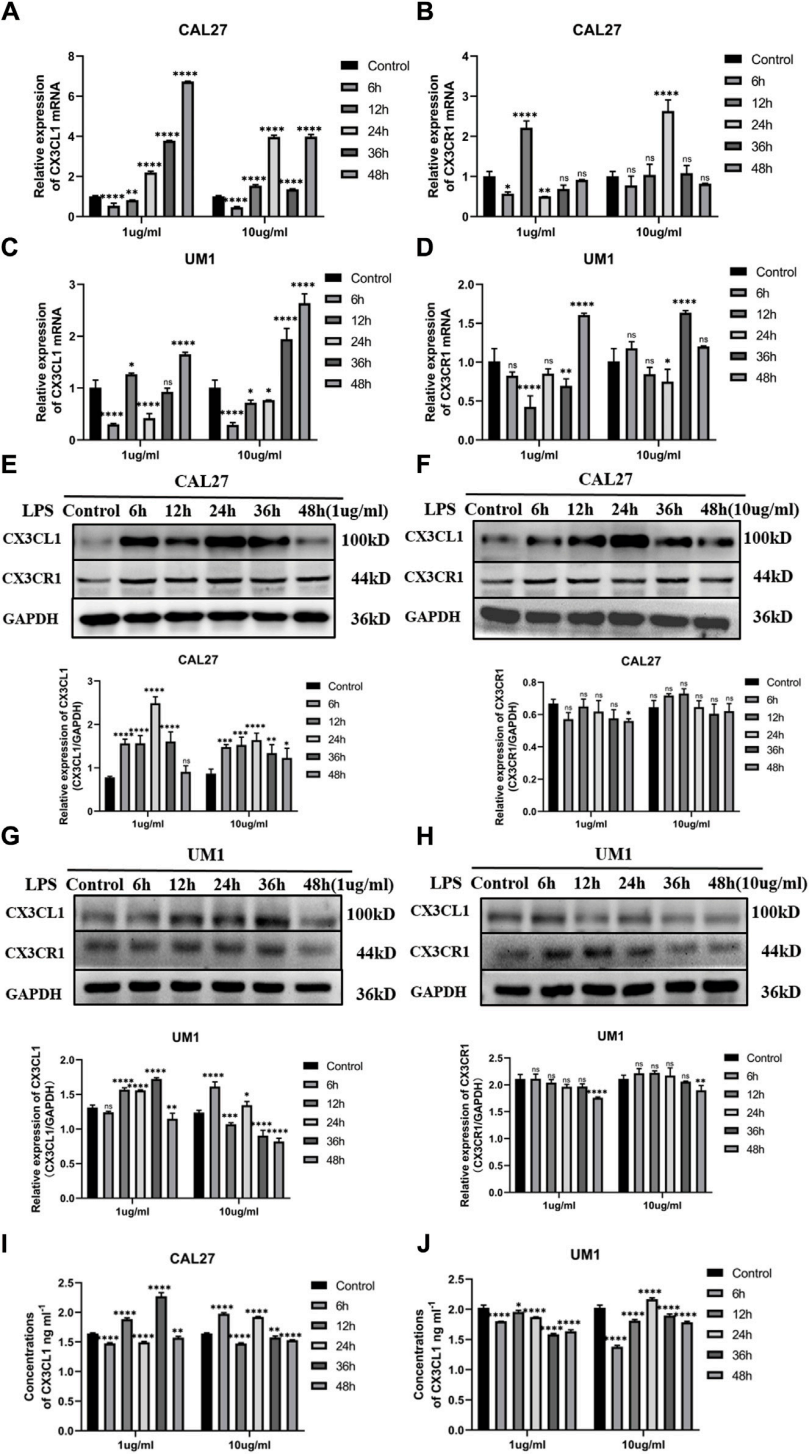
Changes in mRNA and protein expression of *CX3CL1* and *CX3CR1* in CAL27 and UM1 cells before and after LPS stimulation and shows the changes in soluble *CX3CL1* protein concentration in CAL-27 and UM1 cell lines stimulated with 1 μg/mL and 10 μg/mL LPS compared to the control group. Notes: **(A)** and **(B)** show changes in mRNA expression levels of *CX3CL1* and *CX3CR1*, respectively, in CAL27 cells after stimulation with 1 μg/mL and 10 μg/mL LPS compared to the control group treated with an equal volume of sterile PBS. **(C)** and **(D)** show changes in mRNA expression levels of *CX3CL1* and *CX3CR1*, respectively, in UM1 cells after stimulation with 1 μg/mL and 10 μg/mL LPS compared to the control group treated with an equal volume of sterile PBS. **(E)** and **(F)** show the protein band images and grayscale analysis of *CX3CL1* in CAL27 cells after stimulation with 1 μg/mL and 10 μg/mL LPS compared to the control group treated with an equal volume of sterile PBS. **(G)** and **(H)** show the protein band images and grayscale analysis of *CX3CL1* in CAL27 cells after stimulation with 1 μg/mL and 10 μg/mL LPS compared to the control group treated with an equal volume of sterile PBS. **(I)** and **(J)** ELISA shows changes in soluble *CX3CL1* protein concentration in CAL27 and UM1 cell lines after stimulation with 1 μg/m L and 10 μg/mL LPS compared to the control group treated with an equal volume of sterile PBS. Data were shown as mean with SD (n ≥ 3). ns p > 0.05, *p < 0.05, **p < 0.01, ***p < 0.001, ****p < 0.0001.

PCR analysis showed that compared with the control group treated with the same volume of sterile PBS, the mRNA expression of *CX3CL1* was significantly increased in both CAL27 and UM1 cells stimulated with 1 μg/mL and 10 μg/mL LPS (Figures 3A,C), while *CX3CR1* expression remained largely unchanged (Figures 3B,D).

Western blotting analysis showed that compared with the control group treated with the same volume of sterile PBS, the protein expression levels of *CX3CL1* were significantly increased in both CAL27 and UM1 cells stimulated with 1 μg/mL and 10 μg/mL LPS (Figures 3E,G), while *CX3CR1* expression remained largely unchanged (Figures 3F,H). Notably, the protein expression of *CX3CL1* in CAL27 cells stimulated with 1 μg/mL LPS reached its peak at 24 h, while in UM1 cells stimulated with 1 μg/mL LPS, it reached its peak at 36 h, compared with the control group treated with the same volume of sterile PBS.

The results show that after 1 μg/mL and 10 μg/mL LPS stimulation, the mRNA and protein expression of *CX3CL1* in CAL27 cells significantly increased compared to the control group, with the peak protein expression at 24 h after 1 μg/mL LPS stimulation, while the protein content of *CX3CR1* did not change significantly.

ELISA analysis showed that soluble *CX3CL1* was increased in CAL27 cells and decreased in UM1 cells after LPS stimulation compared with the control group treated with the same volume of sterile PBS (Figures 3I,J).

The results show that after stimulation with 1 μg/mL and 10 μg/mL LPS, the mRNA and protein levels of *CX3CL1* in UM1 cells were significantly increased compared to the control group, especially at 36 h after 1 μg/mL LPS stimulation, where the protein expression reached its peak, and the differential expression is statistically significant. However, the protein level of *CX3CR1* did not show significant changes.

### 3.3 Effect of LPS stimulation and overexpression of *CX3CL1* protein on the biological characteristics of OSCC cell lines

Construction of lentiviral overexpression vectors for *CX3CL1* gene was performed using CAL27 and UM1 cell lines. Lentiviral transduction and puromycin selection were used to obtain a transduction efficiency of 80%, as confirmed by fluorescence microscopy. Successful overexpression of *CX3CL1* gene in CAL27 and UM1 cell lines was confirmed by PCR, ELISA and WB. CAL27, UM1 overexpression *CX3CL1* gene were set up in the wild, null, and experimental groups, respectively, abbreviated CAL27^−WT^, CAL27^−GFP^, CAL27^−*CX3CL1*^ UM1^−WT^, UM1^−GFP^, UM1^−*CX3CL1*^. Then LPS was used to stimulate and perform cell proliferation experiment (CCK-8), flow cytometry analysis, Transwell invasion experiment, scratch experiment and colony formation experiment. These experiments were used to determine the effects of *CX3CL1* overexpression on OSCC cell proliferation, cell cycle, invasion, migration, and colony formation ability, as well as the potential reversal effect of LPS stimulation.

#### 3.3.1 Establishment of *CX3CL1* overexpressing cell lines

**FIGURE 4:**
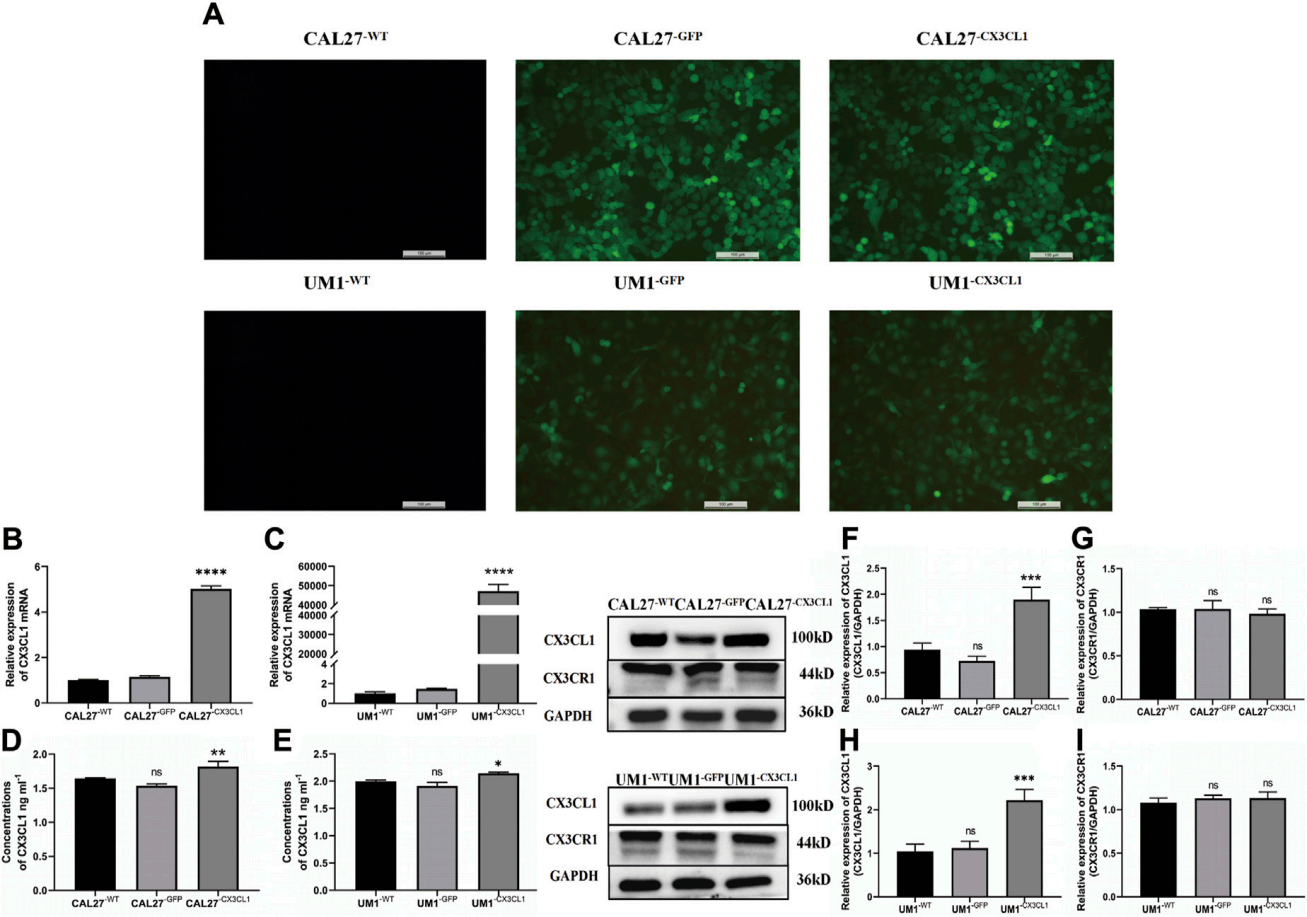
GFP expression in CAL27 and UM1 cell lines detected by fluorescence microscopy and mRNA and protein expression levels of *CX3CL1* and CX3CR1 in CAL27 and UM1 cell lines in each group. Note: **(A)** show that CAL27^−WT^ and UM1^−WT^ showed negative GFP expression, while CAL27^−GFP^, CAL27^−CX3CL1^, UM1^−WT^, and UM1^−GFP^ showed positive GFP expression, with an efficiency of over 80%. **(B)** and **(C)** show the mRNA expression levels of *CX3CL1* in CAL27 and UM1 cells in the experimental group compared with the control and empty vector groups. **(D)** and **(E)** show the changes in soluble *CX3CL1* protein concentration in CAL27 and UM1 cells after overexpression of *CX3CL1*. **(F–I)** show the protein band and gray value analysis of *CX3CL1* and *CX3CR1* in CAL27 and UM1 cells after overexpression of *CX3CL1* compared with the control and empty vector groups. Results showed that the *CX3CL1* mRNA and protein levels were significantly increased in the experimental group compared with the control and empty vector groups, while *CX3CR1* protein content remained unchanged. Data were shown as mean with SD (n ≥ 3).ns, p > 0.05; *, p < 0.05; **, p < 0.01; ***, p < 0.001; ****, <0.0001.

After slow virus transduction and puromycin screening, CAL27 and UM1 cell lines exhibited green fluorescent protein (GFP) expression efficiency of 80% as determined by fluorescence microscopy, indicating a transduction efficiency of 80% (Figure 4A). Subsequently, we verified the expression of *CX3CL1* mRNA and protein using PCR, ELISA, and WB. The control and empty vector groups of CAL27 and UM1 showed no significant changes in *CX3CL1* mRNA and protein expression. In contrast, *CX3CL1* mRNA and protein expression was significantly increased in the CAL27^−*CX3CL1*^ and UM1^−*CX3CL1*^ experimental groups, and the expression of its receptor, *CX3CR1*, showed no significant change, with statistically significant differences (Figures 4B–I). These results indicate that the construction of CAL27 and UM1 cell lines overexpressing *CX3CL1* was successful and can be used for subsequent experiments.

#### 3.3.2 The effect of LPS stimulation and overexpression of *CX3CL1* gene on the biological characteristics of OSCC cell lines

In this study, various groups of CAL27 and UM1 cell lines previously constructed were used to conduct cell proliferation experiments (CCK-8 assay), flow cytometry cell cycle experiments, Transwell invasion experiments, cell scratch experiments, and cell cloning. LPS was used at a concentration and time point corresponding to the peak relative expression of *CX3CL1* protein. Each group of CAL27 cells was stimulated with 1 μg/mL LPS for 24 h, while each group of UM1 cells was stimulated with 1 μg/mL LPS for 36 h before being collected for experiments.

**FIGURE 5:**
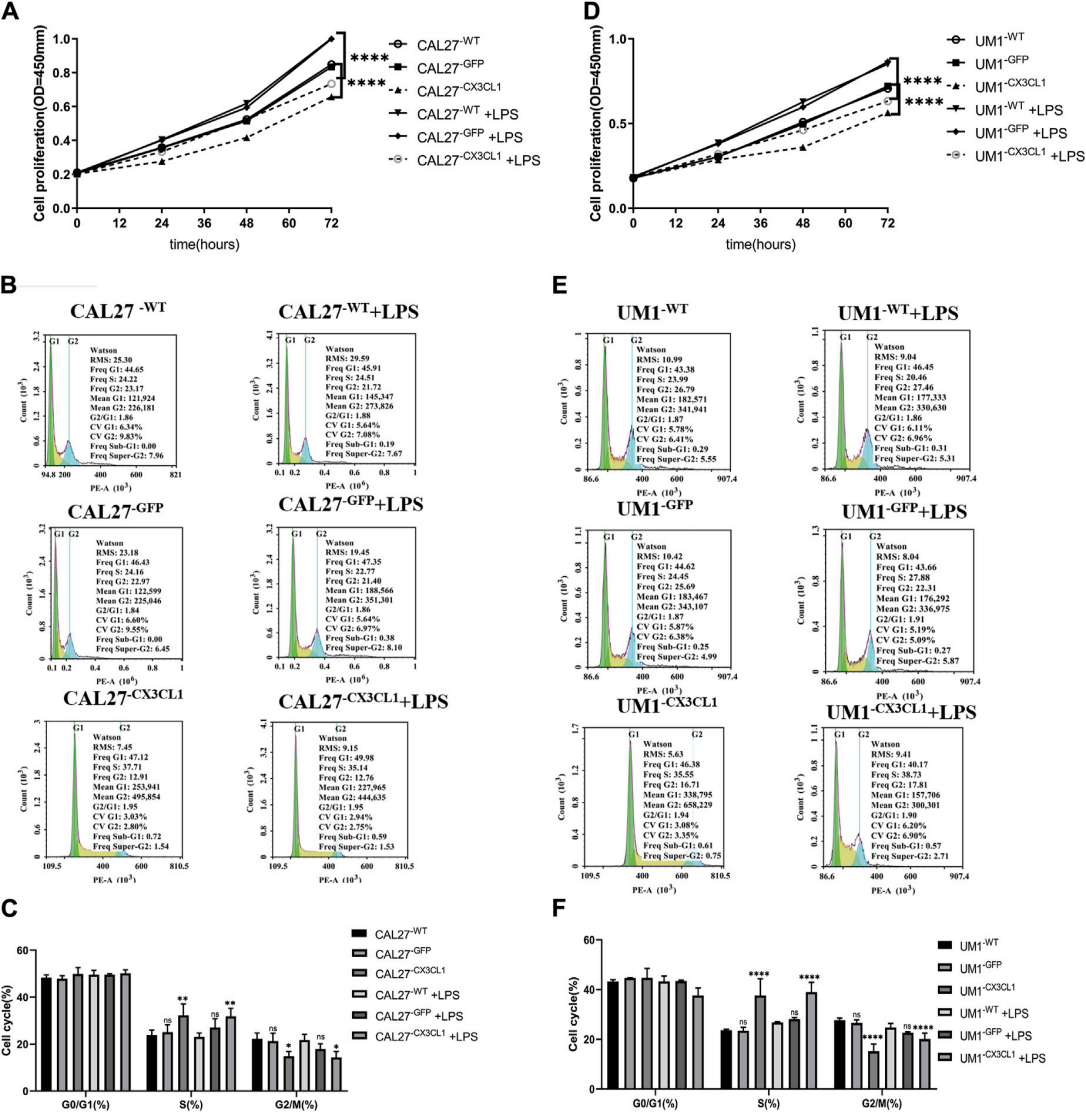
Cell proliferation and cell cycle effects of LPS stimulation and overexpression of *CX3CL1* gene in CAL27 and UM1 cell lines. Note: **(A)** and **(D)** show the changes in OD values of cell proliferation assay in CAL27 and UM1 control group, empty vector group, and experimental group stimulated with 1 μg/mL LPS compared to the addition of the same volume of sterile PBS. **(B)** and **(E)** show the proportion of cell cycle phases during proliferation determined by flow cytometry analysis of CAL27 and UM1 cell lines stimulated with 1 μg/mL LPS. **(C)** and **(F)** show the statistical analysis based on the proportion of cell cycle phases during proliferation of CAL27 and UM1 cell lines in different groups. Results indicate that the experimental group of CAL27 and UM1 cell lines exhibited reduced OD values and cell cycle arrest at S phase compared to the control and empty vector groups, while stimulation with 1 μg/mL LPS increased cell proliferation in CAL27 and UM1 cell lines, but had no significant effect on the cell cycle. Data were shown as mean with SD (n ≥ 3).ns p > 0.05, *p < 0.05, **p < 0.01, ***p < 0.001, ****p < 0.0001.

**FIGURE 6:**
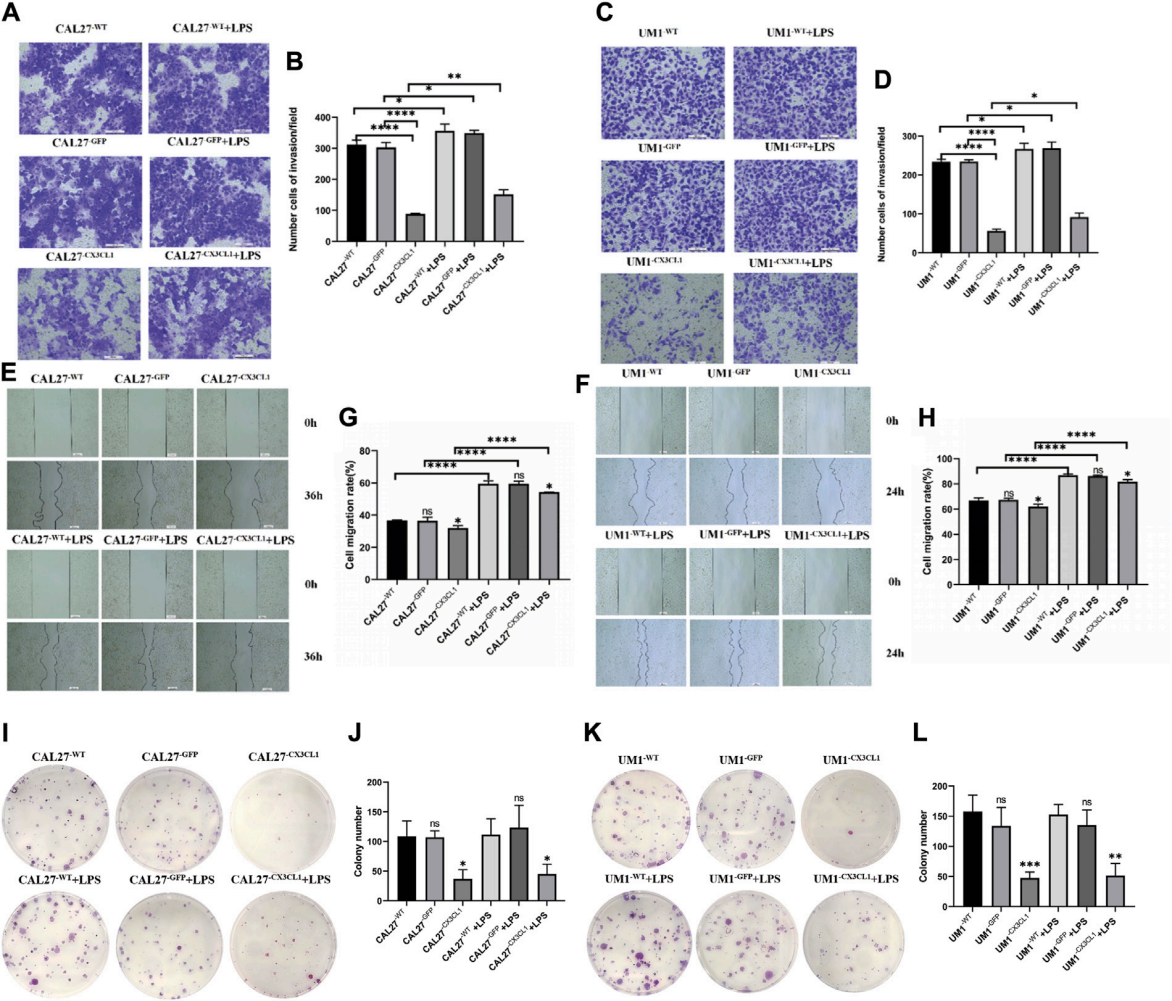
The changes in invasion and migration of CAL27 and UM1 cell lines before and after LPS stimulation and the effect of LPS stimulation on the cloning ability of CAL27 and UM1 cell lines. Note: **(A)** and **(C)** depict the cell invasion of the control group, empty vector group, and experimental group of CAL27 and UM1 cell lines stimulated with 1 μg/mL LPS compared to the same volume of sterile PBS. **(B)** and **(D)** show the statistical analysis of the number of cells passing through the upper chamber of each group of CAL27 and UM1 cell lines. **(E)** and **(G)** depict the cell migration of the control group, empty vector group, and experimental group of CAL27 and UM1 cell lines stimulated with 1 μg/mL LPS compared to the same volume of sterile PBS. **(F)** and **(H)** show the statistical analysis of the change in scratch area of each group of CAL27 and UM1 cell lines. **(I)** and **(K)** show the cloning of the control group, empty vector group, and experimental group of CAL27 and UM1 cell lines stimulated with 1 μg/mL LPS compared to the same volume of sterile PBS. **(J)** and **(L)** show the statistical analysis of the number of clone cell clusters formed by each group of CAL27 and UM1 cell lines. The results show that the number of invaded cells and the change in scratch area of the experimental group of CAL27 and UM1 cell lines decreased compared to the control group and empty vector group. Moreover, the number of invaded cells and the change in scratch area of CAL27 and UM1 cell lines after LPS stimulation increased compared to the same volume of sterile PBS. The results show that the number of clone cell clusters of the experimental group of CAL27 and UM1 cell lines increased compared to the control group and empty vector group. Moreover, there was no significant difference in the number of clone cell clusters of CAL27 and UM1 cell lines after LPS stimulation compared to the same volume of sterile PBS. Data were shown as mean with SD (n ≥ 3).ns p > 0.05, *p < 0.05, **p < 0.01, ***p < 0.001, ****p < 0.0001.

(1) The CCK-8 assay showed that overexpression of *CX3CL1* can inhibit cell proliferation, leading to cell division arrest in the S phase. LPS stimulation promotes OSCC proliferation and overexpression of *CX3CL1* before and after stimulation still promotes cell proliferation without affecting the cell cycle. However, overexpression of *CX3CL1* can partially reverse the promoting effect of LPS on tumor proliferation (Figure 5).
(2) The over expression of *CX3CL1* was verified to inhibit cell invasion and migration using Transwell and scratch assays, while the addition of LPS stimulation can promote the migration and invasion of OSCC cells. (Figures 6A–H).
(3) The cloning experiment verified that the expression of *CX3CL1* can inhibit cell cloning ability, and LPS stimulation has no significant effect on the cloning ability of OSCC (Figures 6I–L).

### 3.4 The effects of LPS stimulation and overexpression of *CX3CL1* protein on AKT activation and expression of epithelial-mesenchymal transition (EMT)-related proteins

Based on the results of previous experiments, both LPS stimulation and overexpression of *CX3CL1* gene had significant effects on the invasion and migration of OSCC cell lines. To explore the underlying mechanisms of these effects, Western blotting was performed to detect the levels of AKT, p-AKT, the epithelial cell marker E-cadherin, the mesenchymal cell markers N-cadherin and Vimentin, and the EMT-related transcription factor Snail protein in various groups of CAL27 and UM1 cells before and after LPS stimulation.

**FIGURE 7:**
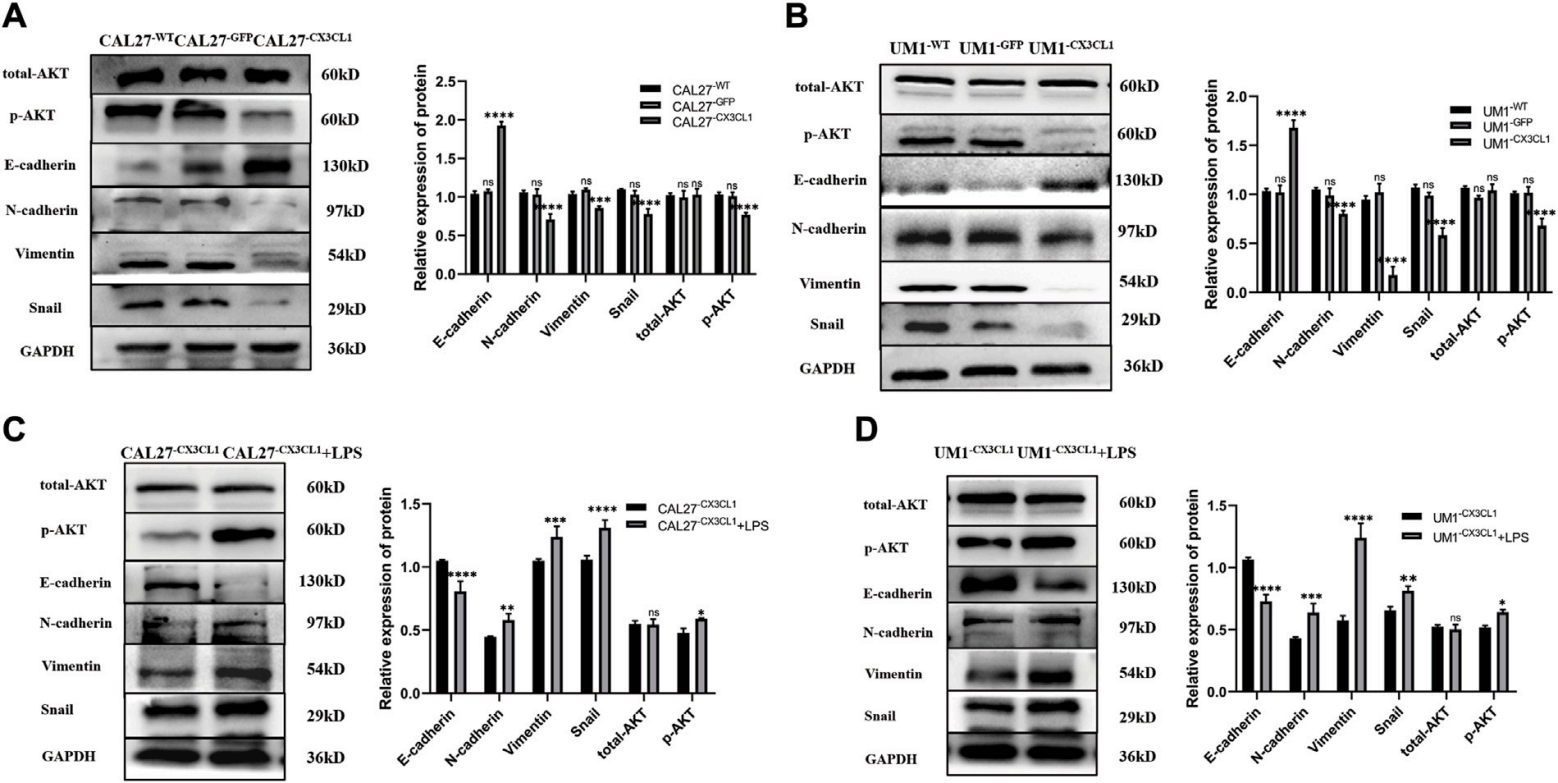
Expression levels of EMT-related and AKT proteins in CAL27 and UM1 cell lines from each group and expression levels of EMT-related proteins and AKT before and after LPS stimulation in *CX3CL1*-overexpressing OSCC cell lines. Note: **(A)** and **(B)** show the protein band display and relative grayscale value analysis of E-cadherin, N-cadherin, Vimentin, Snail, AKT, and p-AKT in CAL27 and UM1 cell lines from each group. Panels **(C)** and **(D)** show the protein band images and relative gray values of E-cadherin, N-cadherin, Vimentin, Snail, AKT, and p-AKT in CAL27 and UM1 experimental groups stimulated with 1 μg/mL LPS compared to those with the same volume of sterile PBS. The results show that the expression of epithelial-related protein E-cadherin increased, while the expression of mesenchymal-related proteins N-cadherin, Vimentin, and Snail decreased. Total AKT did not change significantly, while phosphorylated p-AKT expression decreased significantly, and the expression differences were statistically significant. The results also indicate that E-cadherin expression decreased, while N-cadherin, Vimentin, and Snail expression increased in the stimulated groups. Total AKT expression did not change significantly, while phosphorylated p-AKT expression increased significantly. The expression differences were statistically significant.Data were shown as mean with SD (n ≥ 3). ns p > 0.05, *p < 0.05, **p < 0.01, ***p < 0.001,****p < 0.0001.

The results showed that overexpression of *CX3CL1* gene inhibited AKT activation and EMT-related protein expression, thereby suppressing the biological characteristics of OSCC (Figures 7A, B). On the other hand, LPS stimulation promoted AKT activation and EMT-related protein expression in OSCC cell lines (Figures 7C, D).

## 4 Discussion

Oral squamous cell carcinoma (OSCC) is one of the most common malignant tumors in the head and neck region (Conroy and Lysaght, 2020; Rivas-Fuentes et al., 2021). Due to its high incidence and strong tendency to recur, the 5-year survival rate of OSCC patients is only below 50% (Bugshan and Farooq, 2020; Ling et al., 2021). Surgery is the main treatment with chemotherapy and radiation therapy as adjuvant therapies, which seriously harm the physical and mental health of OSCC patients and greatly reduce their quality of life. Therefore, early detection, diagnosis, and treatment are crucial to reduce the incidence and mortality of OSCC. Thus, the search for new targets for OSCC has important significance (Ohta et al., 2005).

Yang et al. (Cheng-Ning et al., 2015) found that microRNA-29b promotes OSCC migration by downregulating *CX3CL1* expression, and that *CX3CL1* expression levels are negatively correlated with lymph node metastasis and early tumor stage in OSCC patients, suggesting that *CX3CL1* has anti-cancer effects in OSCC. However, more recently, Son et al., 2021 found that TGF-β reduces soluble *CX3CL1*, which in turn affects OSCC bone invasion and improves patient survival, indirectly suggesting that *CX3CL1* also has pro-cancer effects in OSCC. Therefore, in order to investigate the role of *CX3CL1* in OSCC and the effect of lipopolysaccharide (LPS)-induced inflammation on *CX3CL1*, we conducted this study.

This study revealed that *CX3CL1* has an anti-cancer effect in OSCC and that the pro-cancer effect of LPS on *CX3CL1* overexpression has a partial reversal effect. Firstly, bioinformatics analysis, immunohistochemistry, PCR, ELISA, and WB were used to confirm that *CX3CL1* and its receptor *CX3CR1* are lowly expressed in OSCC, while soluble *CX3CL1* is highly expressed, indicating that *CX3CL1* and *CX3CR1* may play an important role in the occurrence and development of OSCC. *CX3CL1* exists in two forms in the human body, membrane-bound and soluble. Membrane-bound *CX3CL1* is located on the cell membrane and mainly acts as an adhesion molecule to enhance cell adhesion, while soluble *CX3CL1* is mainly released into the stroma, and attracts *CX3CR1*-expressing cytotoxic T cells, monocytes, and NK cells to migrate towards the tumor, achieving an anti-tumor effect. Chemokines belong to a category of factors related to inflammation, and inflammation stimulated by LPS has a significant impact on cancer progression, the mechanism of which is not yet clear (Do et al., 2020). Therefore, this study continues to explore the relationship between LPS, *CX3CL1*, and OSCC.

However, compared with the control group treated with equal volume of sterile PBS, LPS stimulation resulted in an increase in *CX3CL1* mRNA and protein expression in CAL27 and UM1 cell lines, with no significant change in *CX3CR1* expression. It has been extensively reported that LPS stimulation significantly enhances the biological characteristics of cancer growth and metastasis, including in OSCC, and is considered a promoting factor for cancer. The relationship between inflammation and cancer development is complex and closely related, with inflammation having both anti-cancer and pro-cancer effects on cancer development, depending on the duration of the inflammation. Therefore, our study found that the anti-cancer effect of *CX3CL1* on OSCC does not conflict with the increased production of *CX3CL1* in LPS-stimulated OSCC.

Next, successful construction of slow virus-mediated overexpression of *CX3CL1* gene in CAL27 and UM1 cell lines was achieved. Biological characteristics were then assessed through some experiments. Results demonstrated that overexpression of *CX3CL1* inhibited the proliferation of OSCC cells, especially in the S phase, and suppressed invasion, migration, and stemness. Moreover, overexpression of *CX3CL1* partially reversed the pro-cancer effect of LPS on OSCC.

Finally, in this study, we explored the mechanism by which overexpression of *CX3CL1* promotes OSCC invasion and migration. We found that the activation of AKT and Epithelial-mesenchymal Transition (EMT) were partially inhibited by overexpression of *CX3CL1*, and that the addition of LPS stimulation led to the promotion of EMT and AKT activation in OSCC cell lines. Studies have shown that AKT activation is closely related to elevated EMT in many cancers, such as colorectal cancer (Ma et al., 2020), cervical cancer (Xu et al., 2021), and bladder cancer (Chi et al., 2022). Our results demonstrate that the increase in *CX3CL1* expression can reduce AKT activation, indicating that *CX3CL1* may regulate EMT by activating AKT to reduce its expression.

**FIGURE 8:**
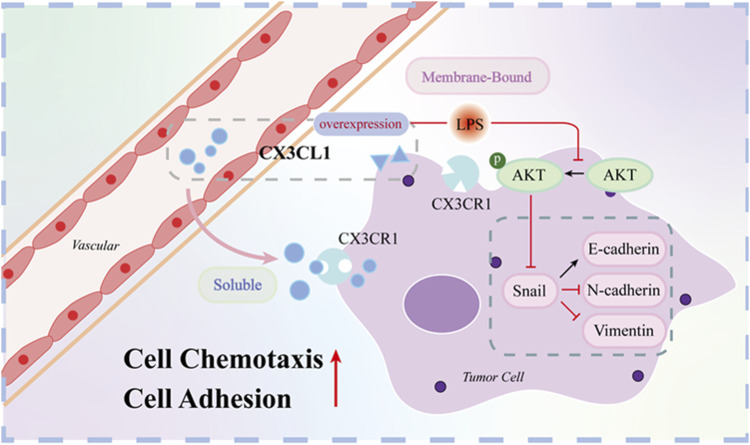
Mechanistic map of *CX3CL1* inhibition of AKT activation and EMT-related protein expression. Note: Figure 8 shows that CX3CL1 is available in membrane-bound and soluble forms. Overexpression of CX3CL1 inhibits AKT activation and EMT-related protein expression. Membrane-bound CX3CL1 is located on the cell membrane and mainly acts as an adhesion molecule to enhance cell adhesion, while soluble CX3CL1 is mainly released into the stroma, and attracts CX3CR1-expressing cytotoxic T cells, monocytes, and NK cells to migrate towards the tumor, achieving an anti-tumor effect. After LPS stimulation, p-AKT and EMT related proteins were changed correspondingly.

Based on the above experimental results, *CX3CL1* exhibits an anti-tumor effect in OSCC. Although the expression of *CX3CL1* mRNA and protein in OSCC is upregulated under the pro-carcinogen LPS stimulus, overexpression of *CX3CL1* can inhibit the biological characteristics and EMT progression of OSCC, and partially reverse the pro-carcinogenic effect of LPS on OSCC (Figure 8).

Therefore, the *CX3CL1* gene is expected to become a new therapeutic target for OSCC. As the specific mechanism by which the *CX3CL1* gene affects OSCC is not yet clear, future studies will continue to explore this gene in more detail. Soluble *CX3CL1* will be studied in the future.

In this study, we investigated the role and mechanism of *CX3CL1* in OSCC. We successfully constructed CAL27 and UM1 cell lines overexpressing *CX3CL1* using lentivirus transduction and performed biological characterization, cell cycle analysis, Transwell migration assay, and scratch assay. We found that *CX3CL1* inhibited proliferation, particularly in the S phase, invasion and migration, and stemness of OSCC cells. Furthermore, we discovered that *CX3CL1* affected the biological characteristics of OSCC by influencing the EMT process and AKT activation, and partially reversed the pro-cancer effect of LPS-induced inflammation on OSCC.

Based on these findings, we believe that *CX3CL1* and *CX3CR1* genes play an important role in the progression of OSCC. Therefore, *CX3CL1* may become a promising new target for the treatment of OSCC. Future research can explore the regulatory mechanism of *CX3CL1* and *CX3CR1* genes, as well as their potential roles in OSCC treatment. Moreover, *CX3CL1* can be considered as a potential drug target for treating OSCC, and related drugs can be further developed and clinically applied.

## Data Availability

The datasets presented in this article are not readily available because Not applicable. Requests to access the datasets should be directed to Not applicable.
